# Long-term treatment outcomes of acromegaly patients presenting biochemically-uncontrolled at a tertiary pituitary center

**DOI:** 10.1186/s12902-017-0199-x

**Published:** 2017-08-04

**Authors:** John D. Carmichael, Michael S. Broder, Dasha Cherepanov, Eunice Chang, Adam Mamelak, Qayyim Said, Maureen P. Neary, Vivien Bonert

**Affiliations:** 1 Pituitary Center, Cedars-Sinai Medical Center, 8700 Beverly Blvd, Los Angeles, CA 90048 USA; 2 Partnership for Health Analytic Research, LLC, 280 S. Beverly Dr., Suite 404, Beverly Hills, CA 90212 USA; 3 Health Economics and Outcomes Research, Novartis Pharmaceuticals Corporation, One Health Plaza, East Hanover, NJ 07936 USA; 4 Global Oncology Market Access and Policy, Novartis Pharmaceuticals Corporation, One Health Plaza, East Hanover, NJ 07936 USA

**Keywords:** Patient registry, Acromegaly, Biochemical control, Insulin-like growth factor-1, Treatment

## Abstract

**Background:**

Acromegaly is a rare, slowly progressive disorder resulting from excessive growth hormone (GH) production by a pituitary somatotroph tumor. The objective of this study was to examine acromegaly treatment outcomes during long-term care at a specialized pituitary center in patients presenting with lack of biochemical control.

**Methods:**

Data came from an acromegaly registry at the Cedars-Sinai Medical Center Pituitary Center (center). Acromegaly patients included in this study were those who presented biochemically-uncontrolled for care at the center. Biochemical control status, based on serum insulin-like growth factor-1 values, was determined at presentation and at study end. Patient characteristics and acromegaly treatments were reported before and after presentation by presenting treatment status and final biochemical control status. Data on long-term follow-up were recorded from 1985 through June 2013.

**Results:**

Seventy-four patients presented uncontrolled: 40 untreated (54.1%) and 34 (45.9%) previously-treated. Mean (SD) age at diagnosis was 43.2 (14.7); 32 (43.2%) were female patients. Of 65 patients with tumor size information, 59 (90.8%) had macroadenomas. Prior treatments among the 34 previously-treated patients were pituitary surgery alone (47.1%), surgery and medication (41.2%), and medication alone (11.8%). Of the 40 patients without prior treatment, 82.5% achieved control by study end. Of the 34 with prior treatment, 50% achieved control by study end.

**Conclusions:**

This observational study shows that treatment outcomes of biochemically-uncontrolled acromegaly patients improve with directed care, particularly for those that initially present untreated. Patients often require multiple modalities of treatment, many of which are offered with the highest quality at specialized pituitary centers. Despite specialized care, some patients were not able to achieve biochemical control with methods of treatment that were available at the time of their treatment, showing the need for additional treatment options.

## Background

Acromegaly is a rare, slowly progressive disorder resulting from excessive growth hormone (GH) production by a pituitary somatotroph tumor. GH produces direct metabolic effects and induces hepatic insulin-like growth factor (IGF)-1 production. IGF-1 in turn also contributes to somatic growth and metabolic dysfunction [1]. Acromegaly affects up to 130 individuals per million persons, or approximately 20,000 people in the US, and recent reports indicate that incidence of pituitary tumors is increasing [2, 3]. Because of the slow progression of symptoms, diagnosis may be delayed for many years, with most acromegaly patients diagnosed after age 40 [2, 4–7]. Diagnosis is made clinically on the basis of typical signs and symptoms confirmed with laboratory assessment of GH and/or IGF-1 levels.

Initial treatment is surgery to resect the adenoma, but at least half of the patients require additional treatment [8–10]. First-line pharmacologic treatment usually consists of one of the first generation somatostatin receptor ligands (SRLs) such as octreotide or lanreotide. The goal of treatment is to reduce GH and/or IGF-1 levels to normal. The efficacy of SRL therapy is highly variable, with an average biochemical response rate of approximately 55% across most large series; however, lower response rates of 17-54% have been observed in several recent prospective studies that included only drug naïve patients [11, 12]. If initial pharmacologic therapy fails to achieve biochemical control, strategies for attempting to improve control include switching to or adding a dopamine agonist or pegvisomant, a GH-receptor antagonist; performing further surgery; or proceeding to radiotherapy [8–10].

The need to better understand this rare and complex disease had resulted in initiation of a number of acromegaly registries worldwide (e.g., [13–21]), although only a few such observational center-specific studies have been conducted in the US. [22–26]. The objective of this study was to report treatment patterns and outcomes of acromegaly patients that presented biochemically-uncontrolled for care at a single US pituitary center, based on data from a US acromegaly registry.

## Methods

### Study design and data sources

This study included patients from an observational acromegaly registry at the Pituitary Center at Cedars-Sinai Medical Center (CSMC) (center). The patients were followed by that center over time, some as early as 1985, and the database was periodically updated, resulting in approximately 300 acromegaly patients in the registry. The data in the registry include demographics, past medical and surgical history, symptoms, laboratory values, medications, cardiology and colonoscopy results, pathology, radiology, and surgical and visual field information. All data were abstracted from medical records into the registry database by center investigators. The study was approved by the CSMC Institutional Review Board.

### Study population and follow-up

The current study focused on acromegaly patients who were followed for at least 12 months after initial treatment and those who were not biochemically-controlled on presentation at CSMC. Presenting biochemical control status was determined based on the initial IGF-1 on presentation to the center. IGF-1 ≤ 100% of upper limit of normal (ULN) was defined as “controlled” and >100% ULN as “uncontrolled” [27]. Although all GH and IGF-1 values were recorded in the study, considering the possiblity of discordance between values of GH and IGF-I in different treatment scenarios, to maintain a robust definition of control we opted to rely solely on IGF-I for this analysis [27].

The first visit at the center was defined as the index date, the baseline period was defined as any time before the index date, and the follow-up period was defined as the time from the index date until the last observed IGF-1 test. Patients with no IGF-1 within the first 90 days after the index date were excluded.

### Study measures

Baseline measures included patient characteristics, coexisting hormonal abnormalities, initial acromegaly treatments, and other medication use. Patient characteristics included age at the index date, age at diagnosis, gender, race/ethnicity, presenting biochemical control status, any abnormal finding on the first magnetic resonance imaging (MRI) or computed tomography (CT) (invasion of cavernous sinus; compression of optic chiasm; and carotid artery encasement), and first reported tumor size (microadenoma [<1 cm] vs. macroadenoma [≥1 cm]). Coexisting hormonal abnormalities included hyperprolactinemia, adrenal insufficiency (i.e., use of adrenal replacement [steroids]), gonadal insufficiency (or use of sex steroid replacement), hypothyroidism (or use of thyroid replacement), and use of antihyperglycemic or antihypertensive medications.

We described each patient’s treatment course both during the baseline period and during follow-up. An individual treatment course was defined as the period from the first date of treatment until a different treatment was instituted. If there were no subsequent treatments, the treatment course ended on the last date of the treatment. Each surgical procedure was counted as a different treatment course. Combination treatment was defined as two or more medications used in conjunction for >90 days. Short (<6 month) pre-surgical medical treatment was not counted as a treatment course, nor was subcutaneous octreotide SA for ≤30 days immediately preceding the use of octreotide LAR or lanreotide.

Finally, final biochemical control status was assessed for all study patients, based on the last observed IGF-1 test result at the center and the same definition as the one described above for presenting biochemical control status. The assays used at the center included: GH and IGF-I assays at Nichols Institute Reference Laboratories (San Juan Capistrano, CA) from 1986 to 1994; Esoterix Inc. (Calabasas, CA) from 1994 to 2005; the Nichols Advantage assay at Nichols Institute/Quest Diagnostics (San Juan Capistrano, CA) from 2005 to 2006; and the DPC Immulite 2000 assay (Diagnostic Products Corp., Los Angeles, CA) at Quest Diagnostics from 2006 to present [27]. All GH and IGF-I assays are two-site RIAs, and each was standardized against World Health Organization international standard preparations, with changes in reference preparations made over the years [27].

### Analyses

Baseline characteristics and baseline treatment variables were presented descriptively for two separate acromegaly cohorts: those presenting uncontrolled and untreated versus those presenting uncontrolled and treated. The two cohorts were then further stratified into those that reached final biochemical control and those that did not, for description of treatment during care at the center. Descriptive statistics, including means, standard deviations (SD), medians, and percentages, were estimated for all study measures when applicable, and reported separately for each control status cohort and for all patients. All data transformations and statistical analyses were performed using SAS® version 9.4 (SAS Institute, Cary, NC).

## Results

Of 300 acromegaly patients in the registry, 121 were followed for at least 12 months after initial treatment, and of these only 74 patients presented biochemically uncontrolled at the center and were included in the study. Of these, 40 patients presented untreated (54.1%), and 34 (45.9%) presented after having received at least one prior treatment. The mean (SD) age at diagnosis was 43.2 (14.7) years and was 47.2 (15.6) years at the time of this study. There were 32 (43.2%) female patients, 55 (74.3%) Caucasian patients, 10 (13.5%) Asian patients, 8 (10.8%) Hispanic patients, and one of other race/ethnicity. Median follow-up at the center was 4.9 years. For 65 of 74 patients, data on baseline tumor size was available: 59 (90.8%) patients had macroadenomas and 6 (9.2%) had microadenomas. Abnormal findings on MRI or CT were observed in 40 (54.1%) patients; 10 (13.5%) patients had hypothyroidism, 9 (12.2%) patients gonadal insufficiency, 7 (9.5%) had adrenal insufficiency, and 1 (1.4%) had elevated prolactin. These patient characteristics were distributed relatively similarly by presenting treatment status (Table 1).

**Table 1:** Baseline patient characteristics by presenting status

	Presenting Uncontrolled Without Prior Treatment for Acromegaly *n* = 40 (54.1%)	Presenting Uncontrolled with Prior Treatment for Acromegaly *n* = 34 (45.9%)	All *N* = 74
Age at index date, year^a^			
mean	44.7	50.1	47.2
(SD)	(15.5)	(15.6)	(15.6)
Age at diagnosis, year^b^			
n	35	31	66
mean	43.8	42.5	43.2
(SD)	(15.5)	(14.0)	(14.7)
Female			
n	16	16	32
(%)	(40.0)	(47.1)	(43.2)
Race/ethnicity			
Caucasian			
n	30	25	55
(%)	(75.0)	(73.5)	(74.3)
Asian			
n	5	5	10
(%)	(12.5)	(14.7)	(13.5)
Hispanic			
n	4	4	8
(%)	(10.0)	(11.8)	(10.8)
Other			
n	1	0	1
(%)	(2.5)	(0.0)	(1.4)
Years of follow-up at center			
mean	5.1	8.5	6.7
(SD)	(4.4)	(5.7)	(5.3)
min - max	0.3–23.0	0.2–21.3	0.2–23.0
median	4.2	7.8	4.9
Tumor Size^c^			
n	36	29	65
Macroadenoma			
n	33	26	59
(%)	(91.7)	(89.7)	(90.8)
Microadenoma			
n	3	3	6
(%)	(8.3)	(10.3)	(9.2)
Abnormal finding on MRI or CT			
n	25	15	40
(%)	(62.5)	(44.1)	(54.1)
Hormonal Abnormalities			
Prolactin elevation			
n	0	1	1
(%)	(0.0)	(2.9)	(1.4)
Adrenal insufficiency			
n	4	3	7
(%)	(10.0)	(8.8)	(9.5)
Gonadal insufficiency			
n	5	4	9
(%)	(12.5)	(11.8)	(12.2)
Hypothyroidism			
n	4	6	10
(%)	(10.0)	(17.6)	(13.5)

^a^Index date was defined as the first visit the center;

^b^66 patients had information about age at diagnosis;

^c^65 patients had tumor size available in the patient medical record; percent among non-missing observations

In the cohort with prior treatment, the pre-presentation (baseline) treatment was pituitary surgery alone in 16 (47.1%) patients, surgery and medication in 14 (41.2%) patients, and medication alone in 4 (11.8%) patients. Medications for acromegaly included only somatostatin analogues and dopamine agonists. In addition, 11.8% used antihyperglycemics and 26.5% antihypertensives. In the untreated cohort, 12.5% used antihyperglycemics and 22.5% used antihypertensive medications (Table 2).

**Table 2:** Baseline treatment by presenting status

	Presenting Uncontrolled Without Prior Treatment for Acromegaly *n* = 40 (54.1%)	Presenting Uncontrolled with Prior Treatment for Acromegaly *n* = 34 (45.9%)	All *N* = 74
Treatment Patterns			
No treatment	40	0	40
	(100.0)	(0.0)	(54.1)
Surgery and medication	0	14	14
	(0.0)	(41.2)	(18.9)
Medication only	0	4	4
	(0.0)	(11.8)	(5.4)
Surgery only	0	16	16
	(0.0)	(47.1)	(21.6)
Pituitary surgery	0	30	30
	(0.0)	(88.2)	(40.5)
Pharmacologic treatment	0	18	18
	(0.0)	(52.9)	(24.3)
Somatostatin analogues	0	12	12
	(0.0)	(35.3)	(16.2)
Pasireotide	0	0	0
	(0.0)	(0.0)	(0.0)
Dopamine agonists	0	12	12
	(0.0)	(35.3)	(16.2)
Pegvisomant	0	0	0
	(0.0)	(0.0)	(0.0)
Antihyperglycemic medication	5	4	9
	(12.5)	(11.8)	(12.2)
Insulin	0	1	1
	(0.0)	(2.9)	(1.4)
Antihypertensive medication	9	9	18
	(22.5)	(26.5)	(24.3)

Somatostatin analogues include octreotide LAR, octreotide SA, and lanreotide; dopamine agonists include bromocriptine and cabergoline

At the end of follow up, 33 (82.5%) of 40 patients without prior treatment achieved control and 7 remained uncontrolled (17.5%). Of 34 patients that presented uncontrolled but with prior treatment, 17 (50%) patients achieved control and 17 remained uncontrolled by study end. Patients that remained uncontrolled tended to be older on average than those that reached control, especially those who were uncontrolled and treated at baseline. Overall, a higher proportions of patients that remained uncontrolled had prolactin elevation (4.2% vs. 0%), adrenal insufficiency (16.7% vs. 6%), and hypothyroidism (20.8% vs. 10.0%) (Table 3).

**Table 3:** Patient characteristics by presenting status and final biochemical control status

Final Biochemical Control		Presenting Uncontrolled Without Prior Treatment for Acromegaly *n* = 40 (54.1%)		Presenting Uncontrolled with Prior Treatment for Acromegaly *n* = 34 (45.9%)		All *N* = 74
		Controlled *n* = 33 (82.5%)	Uncontrolled *n* = 7 (17.5%)	Controlled *n* = 17 (50.0%)	Uncontrolled *n* = 17 (50.0%)	
Age at index date, year^a^						
mean		44.5	45.6	46.8	53.4	47.2
(SD)		(15.9)	(14.5)	(12.9)	(17.7)	(15.6)
Age at diagnosis, year^b^						
N		30	5	16	15	66
mean		44.5	40.0	41.4	43.6	43.2
(SD)		(15.9)	(13.6)	(12.1)	(16.1)	(14.7)
Years of follow-up at center						
mean		5.0	5.3	8.7	8.3	6.7
(SD)		(4.7)	(2.7)	(6.3)	(5.3)	(5.3)
min-max		0.3-23.0	2.2-9.4	0.2-21.3	0.6-20.2	0.2-23.0
median		4.2	4.5	8.0	7.3	4.9
Tumor Size^c^						
n		31	5	15	14	65
Macroadenoma						
n		29	4	14	12	59
(%)		(93.5)	(80.0)	(93.3)	(85.7)	(90.8)
Microadenoma						
n		2	1	1	2	6
(%)		(6.5)	(20.0)	(6.7)	(14.3)	(9.2)
Abnormal finding on MRI or CT						
n		21	4	7	8	40
(%)		(63.6)	(57.1)	(41.2)	(47.1)	(54.1)
Hormonal Abnormalities						
Prolactin elevation						
n		0	0	0	1	1
(%)		(0.0)	(0.0)	(0.0)	(5.9)	(1.4)
Adrenal insufficiency						
n		2	2	1	2	7
(%)		(6.1)	(28.6)	(5.9)	(11.8)	(9.5)
Gonadal insufficiency						
n		4	1	4	0	9
(%)		(12.1)	(14.3)	(23.5)	(0.0)	(12.2)
Hypothyroidism						
n		3	1	2	4	10
(%)		(9.1)	(14.3)	(11.8)	(23.5)	(13.5)

^a^Index date was defined as the first visit at the center;

^b^35 patients had information about age at diagnosis in the presenting uncontrolled without prior treatment group; 31 patients had the information in the presenting uncontrolled with prior treatment group;

^c^36 patients had tumor size available in the patient medical record in the presenting uncontrolled without prior treatment group; 29 patients had the information in the presenting uncontrolled with prior treatment group

Among the 33 initially uncontrolled and untreated patients that reached control by study end, most were managed with surgery and medication (51.5%) or surgery alone (42.4%) after presentation. Among the 17 initially uncontrolled but treated patients that reached control by study end, most were managed with medication alone (58.8%) during care at the center (Table 4).

**Table 4:** Treatment during care at center by presenting status and final biochemical control status

Final Biochemical Control	Presenting Uncontrolled Without Prior Treatment for Acromegaly *n* = 40 (54.1%)		Presenting Uncontrolled With Prior Treatment for Acromegaly *n* = 34 (45.9%)		All *N* = 74
	Controlled *n* = 33 (82.5%)	Uncontrolled *n* = 7 (17.5%)	Controlled *n* = 17 (50.0%)	Uncontrolled *n* = 17 (50.0%)	
Treatment Patterns					
Surgery and medication	17	4	2	4	27
	(51.5)	(57.1)	(11.8)	(23.5)	(36.5)
Medication only	2	2	10	12	26
	(6.1)	(28.6)	(58.8)	(70.6)	(35.1)
Surgery only	14	1	3	1	19
	(42.4)	(14.3)	(17.6)	(5.9)	(25.7)
No treatment	0	0	2	0	2
	(0.0)	(0.0)	(11.8)	(0)	(2.7)
Pituitary surgery	31	5	5	5	46
	(93.9)	(71.4)	(29.4)	(29.4)	(62.2)
Pharmacologic treatment	19	6	12	16	53
	(57.6)	(85.7)	(70.6)	(94.1)	(71.6)
Somatostatin analogues	15	4	9	15	43
	(45.5)	(57.1)	(52.9)	(88.2)	(58.1)
Pasireotide	0	0	1	0	1
	(0.0)	(0.0)	(5.9)	(0.0)	(1.4)
Dopamine agonists	8	2	6	10	26
	(24.2)	(28.6)	(35.3)	(58.8)	(35.1)
Pegvisomant	5	1	2	4	12
	(15.2)	(14.3)	(11.8)	(23.5)	(16.2)

Somatostatin analogues include octreotide LAR, octreotide SA, and lanreotide; dopamine agonists include bromocriptine and cabergoline; dopamine agonists include bromocriptine and cabergoline

## Discussion

This study describes the treatment patterns and outcomes of acromegaly patients that presented without biochemical control at a single major specialized pituitary center in the US. The study showed that of those patients that presented at the center biochemically-uncontrolled and previously untreated for acromegaly, a majority (82.5%) achieved biochemical control during care at the center by study end. However, only 50% of patients that presented as biochemically-uncontrolled but had prior treatment went on to achieve biochemical control by study end, suggesting these cases may have been more complex.

Although there are a number of published studies based on acromegaly registries worldwide (e.g., [13–21]), only a few such studies have reported on results for the US acromegaly population [22–26]. The current study supplements the literature by providing a description of treatment patterns based on an ongoing US acromegaly registry.

The results from this study indicate that a higher proportion of acromegaly patients that present de novo*,* uncontrolled and untreated, to a specialized pituitary center may achieve disease remission compared to acromegaly patients that present uncontrolled and previously treated. These data suggest that the patients that are uncontrolled and previously treated at presentation may be more treatment resistant and have more complex management requirements than those that present untreated. It is possible that the previously treated patients are referred to a specialized pituitary center due to failing treatment elsewhere. The complexity of previously treated cases was underscored in this study by a somewhat higher proportion of these patients presenting with use of antihypertensive medications (26.5% vs. 22.5%) and with hypothyroidism (17.6% vs. 10%) indicating a potentially higher degree of hypopituitarism; they were also slightly older (mean age 50.1 versus 44.7 years). The management complexity of these patients was also indicated by an overall longer follow-up time period versus those that presented untreated (median follow-up period of 7.8 years vs. 4.2 years). These patients may have had a longer duration of the disease that was left uncontrolled and untreated, manifesting in higher rates of comorbidities.

Conversely, these results also show that even among uncontrolled patients that previously received treatment for acromegaly, such as pituitary surgery, further disease management at a specialized pituitary center results in improved biochemical control in up to 50% of patients. These improvements are likely associated with access to successful repeat pituitary surgery or specialized tailoring of medical therapy for patients not suited for surgery. Specifically, in this study the use of medical therapies and surgeries, including pegvisomant and repeat surgeries by an experienced neurosurgeon led to an improvement in disease control in most patients presenting to the center. Finally, these center-specific findings suggest that for some difficult-to-treat acromegaly patients, achieving biochemical control even at a specialized pituitary center may be challenging with currently available treatment options.

Patients who remained uncontrolled despite maximal surgical and medical treatment were considered for radiosurgery or radiotherapy. Risks and benefits of these therapies were weighed in all cases, and in all cases presented here, patients did not receive radiation during the treatment period described. In general, the approach towards patients with acromegaly at this center ascribes radiation to a lower treatment priority than surgery or medication, and weighs heavily the risks associated with radiation treatment [9, 10].

This study provided a large data set on acromegaly patients treated at a specialized pituitary center, thereby filling the need for more data on observed long-term treatment outcomes for acromegaly in the US. The study included detailed medical chart information across a lengthy follow-up period, enabling tracking of treatment patterns and associated clinical outcomes. The study had limitations. The small sample size was limited in power to conduct statistical tests to assess significant differences between cohorts in the study sample. It is possible that results vary across centers. Future studies should compare the outcomes of acromegaly patients treated at different centers. Patients examined in this study likely represent cases with complex acromegaly, referred for specialized tertiary or quaternary care. The study reflects care over more than two decades. Acromegaly management has changed significantly over that time. Since this study was completed, some patients may have achieved control. Other limitations include those that are typical of observational studies and registries, such as lack of randomized-placebo controlled study design, which would have ensured strict criteria such as medication titration protocols and treatment adherence.

## Conclusions

Treatment outcomes for biochemically-uncontrolled acromegaly patients improve with directed care, particularly for those that initially present untreated. Patients often require multiple modalities of treatment, many of which are offered at specialized pituitary centers. Despite care at such a center, some patients did not achieve biochemical control with currently available methods of treatment, showing a clear unmet need for additional treatment options. Future research should consider that in any evaluation of a clinical practice, treatment decisions and outcomes are not only guided by physicians’ clinical management decisions and preferences, but also by patients’ access to care (such as insurance), preferences, and treatment compliance.

## Abbreviations

CSMC: Cedars-Sinai Medical Center
GH: Growth hormone
IGF: Insulin-like growth factor
SD: Standard deviations
SRLs: Somatostatin receptor ligands
ULN: Upper limit of normal
